# Supplementary material for: Characterization of Polyphenolic Compounds from Bacopa procumbens and Their Effects on Wound-Healing Process
Source: Molecules. 2022 Oct 2;27(19):6521. doi: 10.3390/molecules27196521 (PMC9571823; doi:10.3390/molecules27196521)
Supplement: Supplementary file 1 [file molecules-27-06521-s001.zip › molecules-1917338-supplementary.pdf]

Supplementary Materials

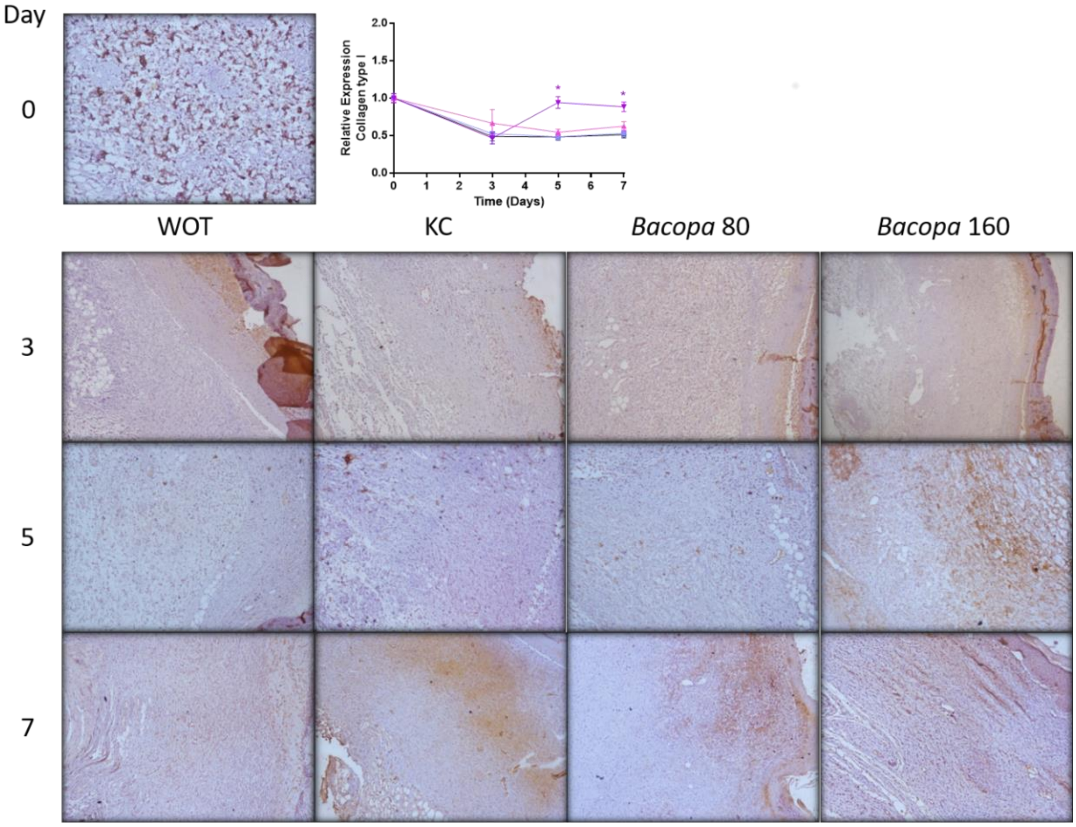

Figure S1. Collagen type I expression. Representative microphotographs of collagen type I protein by immunohistochemistry detection on wound tissue at different periods of time (0, 3, 5 and 7 days) in WOT (black line), KC (blue line), and 80 mg/mL (pink line) and 160 mg/mL (purple line) of *PB*. *$p < 0.05$ vs WOT.

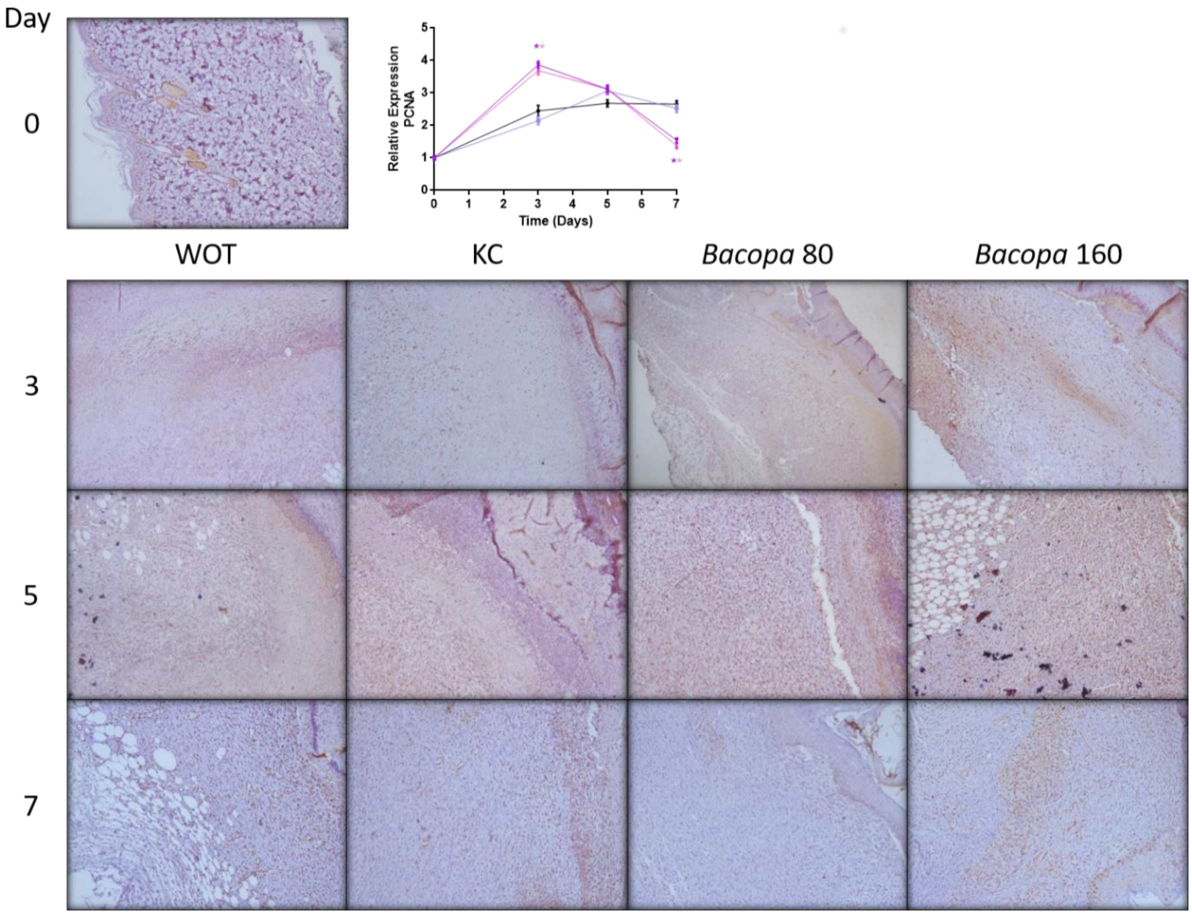

Figure S2. PCNA expression. Representative microphotographs of PCNA protein by immunohistochemistry detection on wound tissue at different periods of time (0, 3, 5 and 7 days) in WOT, WOT (black line), KC (blue line), and 80 mg/mL (pink line) and 160 mg/mL (purple line) of PB. *p < 0.05 vs WOT.

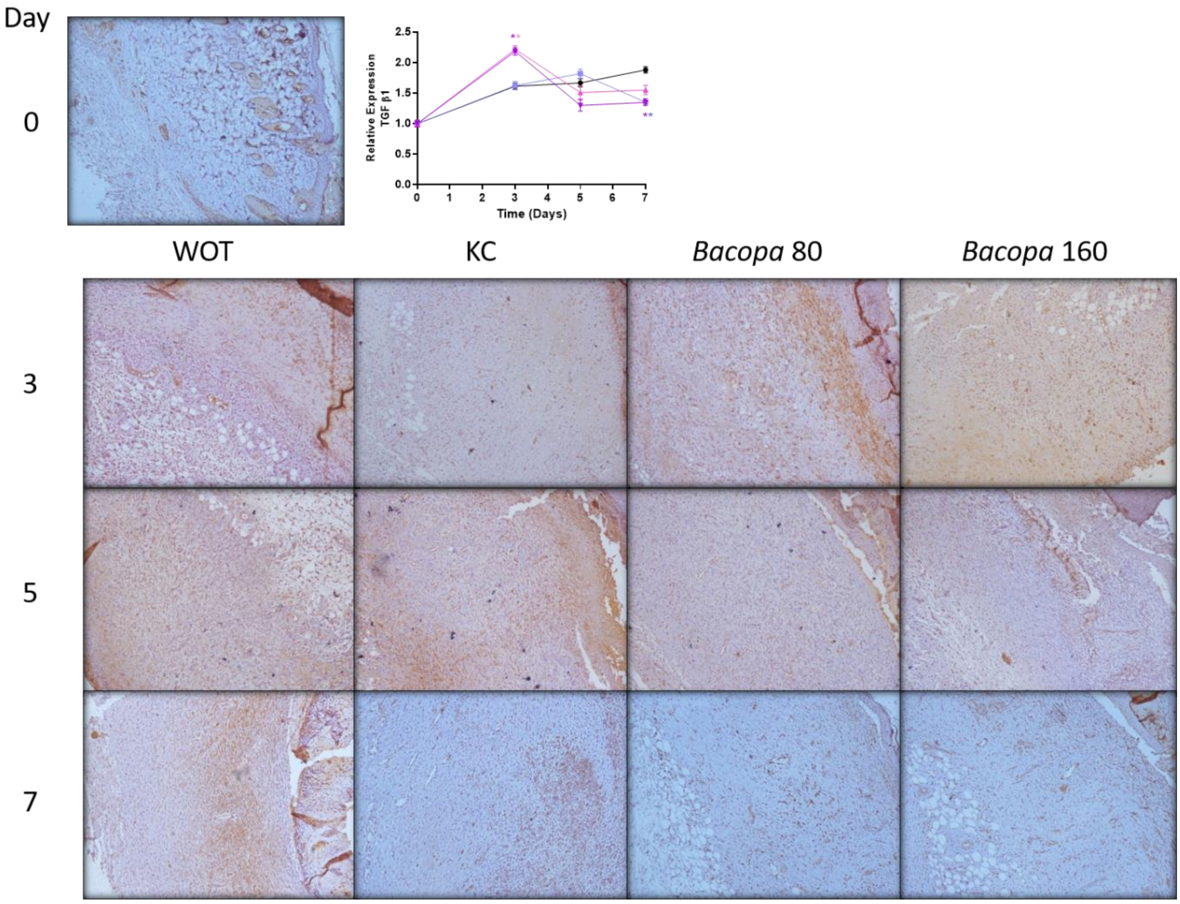

Figure S3. TGFβ1 expression. Representative microphotographs of TGFβ1 protein expression from wound tissues were analyzed by immunohistochemistry at different periods of time (0, 3, 5 and 7 days) in WOT (black line), KC (blue line), and 80 mg/mL (pink line) and 160 mg/mL (purple line) of *PB*. $^{*}p < 0.05$ vs WOT.

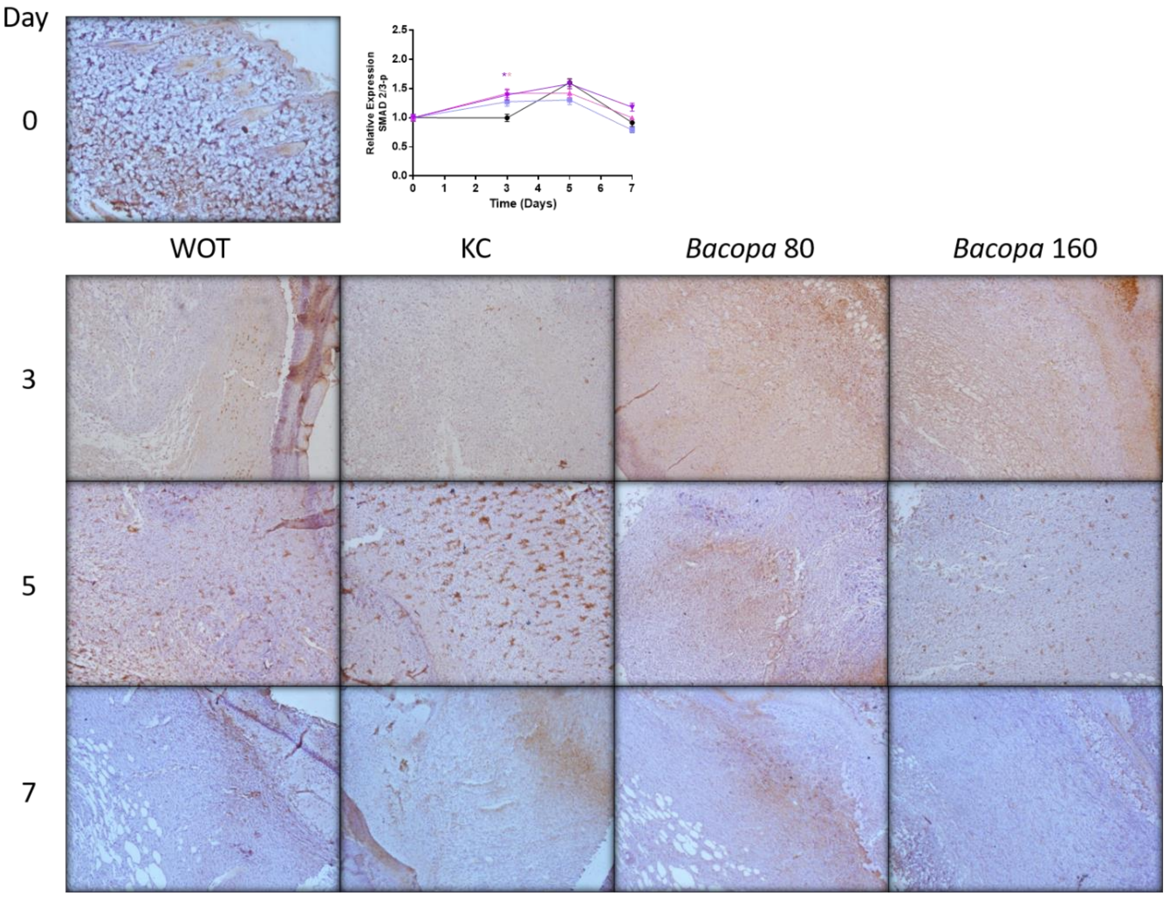

Figure S4. SMAD 2/3-p expression. Representative microphotographs of SMAD 2/3 phosphorylated protein by immunohistochemistry detection on wound tissue at different periods of time (0, 3, 5 and 7 days) in WOT (black line), KC (blue line), and 80 mg/mL (pink line) and 160 mg/mL (purple line) of *PB*. $^{*}p < 0.05$ vs WOT.

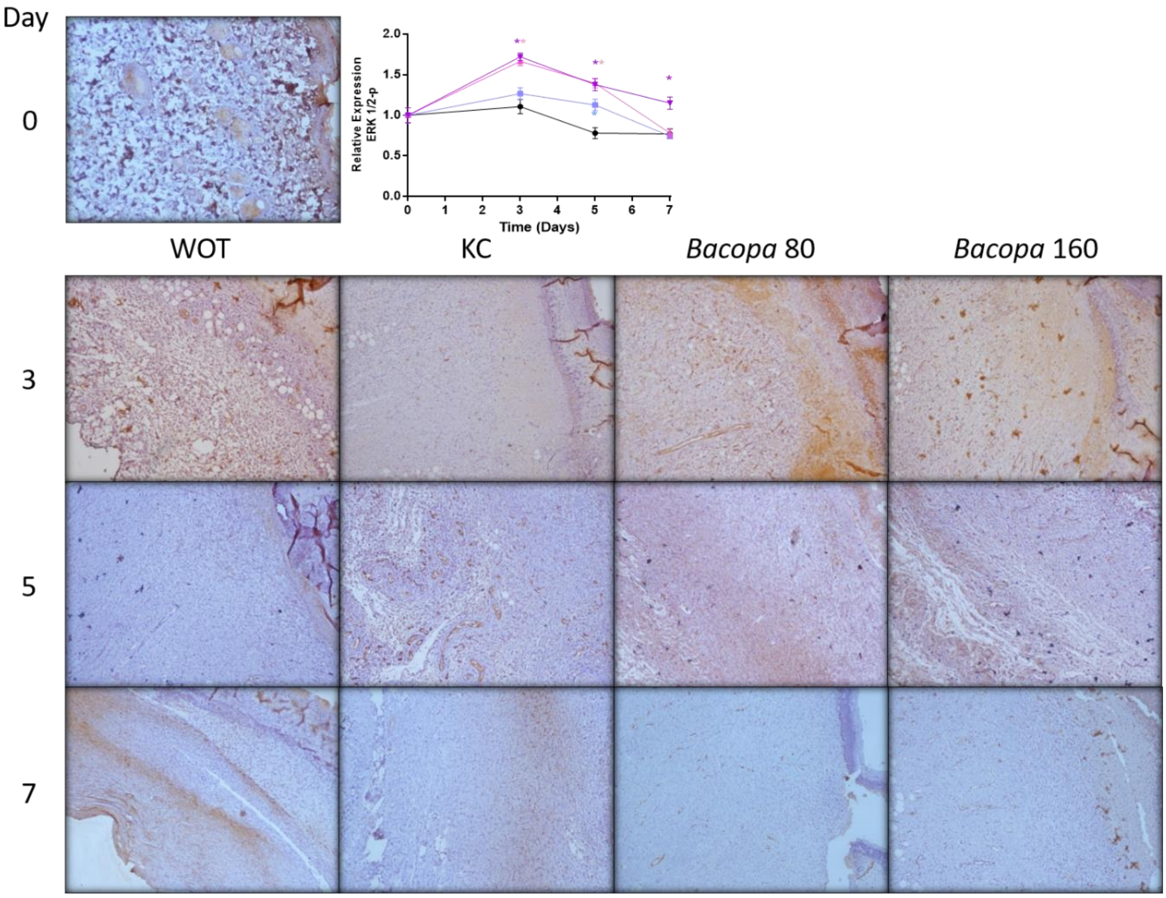

Figure S5. ERK 1/2-p expression. Representative microphotographs of ERK 1/2 phosphorylated protein by immunohistochemistry detection on wound tissue at different periods of time (0, 3, 5 and 7 days) in WOT (black line), KC (blue line), and 80 mg/mL (pink line) and 160 mg/mL (purple line) of *PB*. *$p < 0.05$ vs WOT.
